# Adaptability of a jump movement pattern to a non-constant force field elicited via centrifugation

**DOI:** 10.1371/journal.pone.0230854

**Published:** 2020-04-08

**Authors:** Andreas Kramer, Jakob Kümmel, Maren Dreiner, Steffen Willwacher, Timo Frett, Anja Niehoff, Markus Gruber

**Affiliations:** 1 Department of Sport Science, University of Konstanz, Konstanz, Germany; 2 Institute of Biomechanics and Orthopaedics, German Sports University Cologne, Cologne, Germany; 3 Institute of Aerospace Medicine, German Aerospace Center, Cologne, Germany; 4 Cologne Center for Musculoskeletal Research, Medical Faculty and University Hospital Cologne, University of Cologne, Cologne, Germany; University of Belgrade, SERBIA

## Abstract

Humans are accustomed to Earth’s constant gravitational acceleration of 1g. Here we assessed if complex movements such as jumps can be adapted to different acceleration levels in a non-constant force field elicited through centrifugation. Kinematics, kinetics and muscle activity of 14 male subjects (age 27±5years, body mass 77±6kg, height 181±7cm) were recorded during repetitive hopping in a short-arm human centrifuge for five different acceleration levels (0.5g, 0.75g, 1g, 1.25g, 1.5g). These data were compared to those recorded during normal hops on the ground, and hops in a previously validated sledge jump system. Increasing acceleration from 0.5g to 1.5g resulted in increased peak ground reaction forces (+80%, p<0.001), rate of force development (+100%, p<0.001) and muscle activity (+30 to +140%, depending on phase, side and muscle). However, most of the recorded parameters did not attain the level observed for jumps on the ground or in the jump system. For instance, peak forces during centrifugation with 1g amounted to 60% of the peak forces during jumps on the ground, ground contact time was prolonged by 90%, and knee joint excursions were reduced by 50%. We conclude that in principle, a quick adaptation to acceleration levels other than the normal constant gravitational acceleration of 1g is possible, even in the presence of a non-constant force field and Coriolis forces. However, centrifugation introduced additional constraints compared to a constant force field without rotation, resulting in lower peak forces and changes in kinematics. These changes can be interpreted as a movement strategy aimed at reducing lower limb deflections caused by Coriolis forces.

## Introduction

On earth, everything is subjected to Earth’s constant gravitational acceleration. Whether this leads to a strong adaptation of human movement to a constant acceleration of 1g, or whether humans can quickly adapt to unaccustomed accelerations levels and non-constant force fields, is an open question. The answer to this question is particularly important for astronauts on space missions, as they do not experience this gravitational acceleration, leading to substantial undesirable adaptations to this microgravity environment, such as loss of bone mass, muscle mass and function, aerobic capacity and orthostatic tolerance [1]. Several exercise countermeasures have already been tested, and in a recent study, plyometrics were able to maintain bone mass, muscle mass and function as well as aerobic capacity [2–5]. While preserving the most important aspects of physical performance, the jump training was not able to completely preserve orthostatic tolerance [6], and had no effect on the decrease in blood and plasma volume [4]. Artificial gravity elicited in a human centrifuge however might have the potential to preserve orthostatic tolerance and counteract other systemic effects [7], even though it is unlikely that it will have a substantial effect on muscle and bone mass due to its lack of impact loading [8]. Combining these two countermeasures—plyometrics and artificial gravity elicited via centrifugation—might have the potential to offset all of the negative effects of unloading in space.

In addition to this applied perspective, it is of fundamental interest whether the neuromuscular system is able to adapt to the force field elicited during centrifugation: the first challenge is that the force field during centrifugation is not constant, as the force acting on an object being centrifuged will depend on its distance from the center of rotation. Thus, with the head near the center of rotation and the feet pointing outwards, the force at the feet will be much higher than the force at the torso or the head. The second challenge is the Coriolis effect, i.e., the deflection of a moving object during rotation due to the inertial Coriolis force. When performing jumps along the radial direction during clockwise centrifugation, the feet should be deflected to the left from the jumper’s perspective, making the jump asymmetrical and potentially requiring the neuromuscular system to activate the muscles of the left leg differently than the muscles of the right leg for the deflection to be compensated. The deflection might also lead to higher knee joint moments. The third challenge arises when using rotational speeds that result in higher or lower accelerations than the accustomed gravitational accelerations of 1g. Previous studies using a sledge jump system (SJS) suggest that in a constant force field, the neuromuscular system is able to cope with accelerations other than 1g [9].

To answer the question whether complex whole-body movements such as jumps are possible during centrifugation despite a non-constant force field, the Coriolis effect and accelerations other than 1g, we compared jumps on a short-arm human centrifuge to jumps in a previously validated sledge jump system [10] as well as normal jumps on the ground. We hypothesized that jumps in the centrifuge would differ from normal jumps on the ground as well as from jumps in the SJS with respect to peak ground reaction forces (lower), ground contact times (longer), joint angles (more flexed), knee joint moments (higher for adduction and internal rotation), and preactivity of the leg extensors (lower). In addition, we hypothesized that the acceleration level would influence these parameters, and that there would be differences between the left and the right leg due to the effects of the Coriolis force.

## Methods

### Subjects

Fourteen healthy male subjects volunteered to participate in this study. They were recreationally active, but were naïve to both the centrifuge and the SJS. Being able to consistently jump with good technique was a prerequisite for participating (visually assessed by the authors and confirmed with force plate recordings). The study was restricted to male participants purely for practical reasons (marker placement on delicate landmarks). Their mean (± standard deviation, SD) age, body mass and height were 27±5 years, 77±6kg, and 181±7cm. All participants gave written informed consent to the experimental procedure, which was approved by the ethics committee of the Northern Rhine Medical Association (Ärztekammer Nordrhein) in Duesseldorf, Germany and in accordance with the Declaration of Helsinki. The study was registered in the German Clinical Trial Registry (DRKS00014001).

### Experimental design

**Fig 1:**
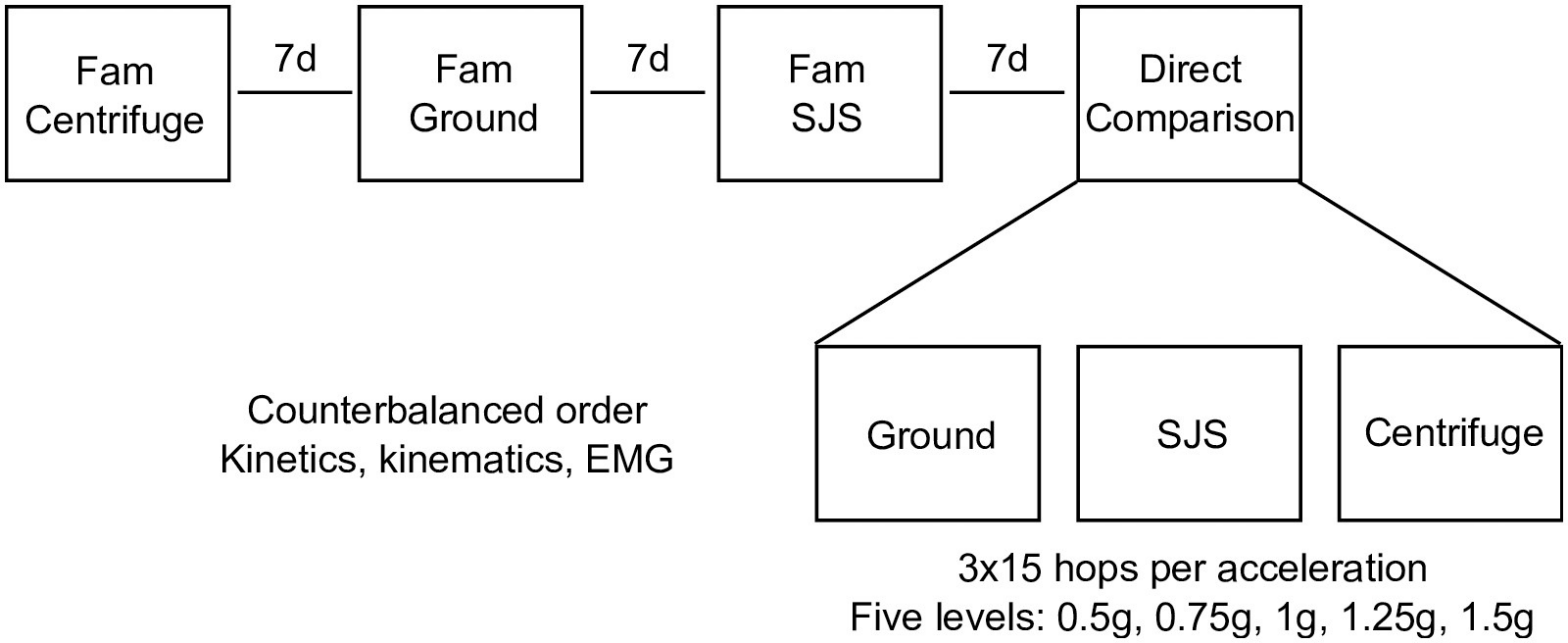
Study design. Before performing the comparison measurements, all participants were familiarized with the three jump modes (centrifuge, ground, and sledge jump system) on three different occasions, with one week in between sessions and counterbalanced order between subjects. Each of the familiarization sessions consisted of 15 series of 15 repetitive hops each, with an acceleration of 1g. During the comparison session (the basis of this paper’s analyses), participants performed three series of 15 hops each per acceleration level, i.e., 15 series in total for the centrifuge, 15 series for the SJS, and three series on the ground. The order of the three conditions was counterbalanced between participants.

**Fig 2:**
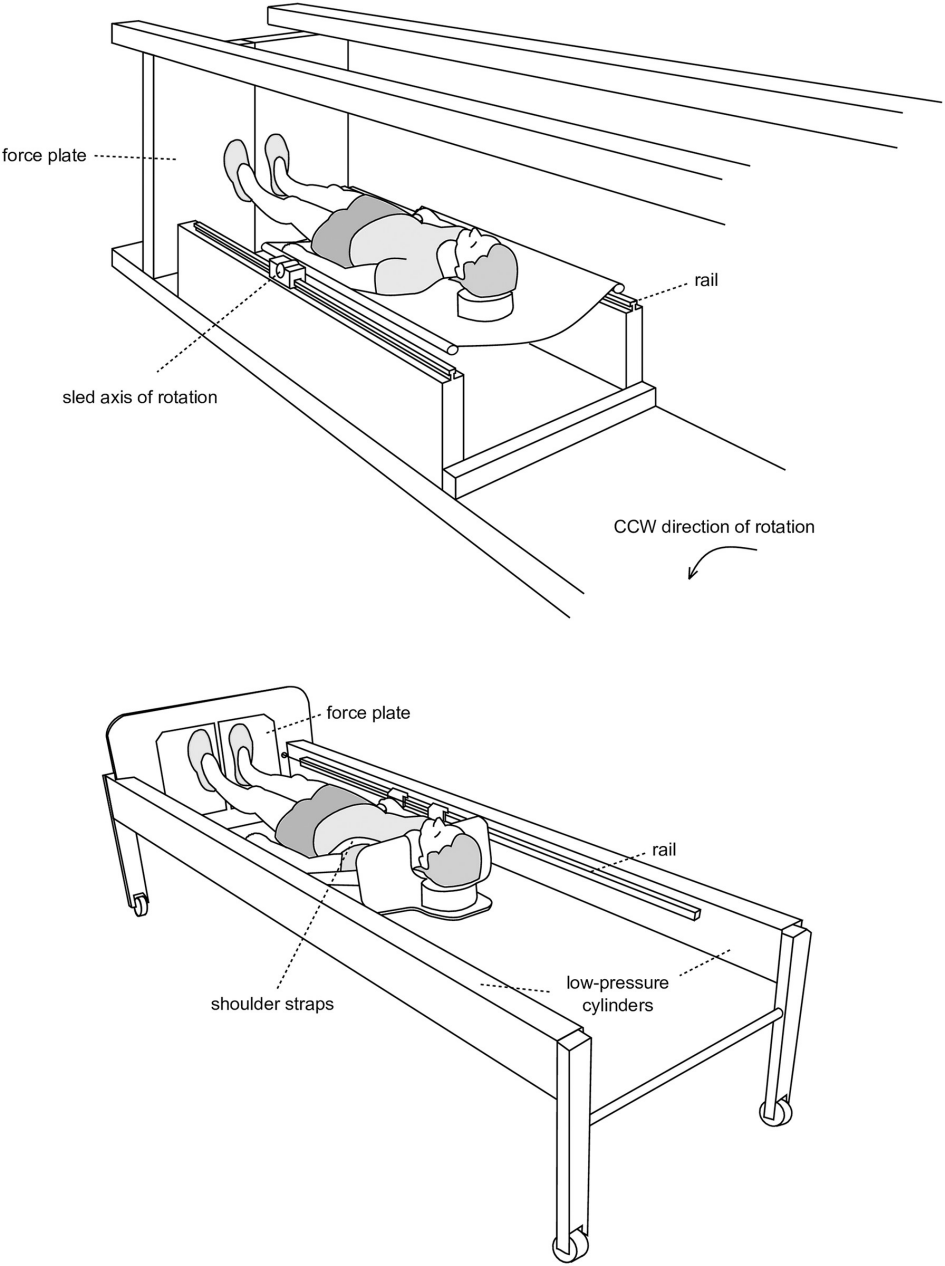
Participant in the short-arm human centrifuge (top) and in the sledge jump system (bottom). In the centrifuge, the participant lies on a sled that can slide along two rails. The sled is fixed at the rails, with the fixation points at waist height serving as a rotational axis, allowing pitch. In the sledge jump system, the participant is fixed to the wooden sledge with straps. The straps are attached to the rails and can slide in the direction of the rails with hardly any restrictions concerning joint angles. Partly adapted from [4].

A single-group repeated-measures study design was used to compare hops in a centrifuge to normal hops on the ground as well as hops in a SJS. In the centrifuge and the SJS, five different accelerations (0.5, 0.75, 1, 1.25, and 1.5g) were used. Before performing the comparison measurements, all participants were familiarized with the three jump modes (ground, centrifuge, SJS) on three different occasions, with one week in between sessions (with the exception of one participant who once had two sessions in one week due to scheduling problems) and counterbalanced order between subjects, see Fig 1. Each of the familiarization sessions consisted of 15 series of 15 repetitive maximal two-leg hops each, with an acceleration of 1g. After a ten-minute warm-up phase (consisting of 10 squats and 10 heel raises), the two-leg hops were performed with the instruction “Jump as stiff as possible, i.e. flex the ankle, knee and hip joint as little as possible while still jumping as high as the high stiffness allows; keep the contact time as short as possible and do not let the heels touch the plate during landing”. Hands were kept akimbo (jumps on the ground) or on handles attached to the sled at waist height, see Fig 2 (jumps in the SJS and the centrifuge). During the comparison session, participants performed three series of 15 hops each per acceleration level, i.e., 15 series in total for the centrifuge, 15 series for the SJS, and three series on the ground. With an average exercise time of 8s per series, this resulted in a total exercise time of about five minutes. The order of the three conditions was counterbalanced between participants, with at least one minute of rest in between series and half an hour in between conditions. The hops were performed bare-footed on two force plates (Leonardo^®^, Novotec Medical, Germany, for the SJS; AMTI, Watertown, USA, for the centrifuge and on the ground). The ground reaction forces (GRF) were recorded separately for the right and the left foot with a sampling frequency of 1000Hz and synchronized to the electromyographic (EMG) signals and the motion capturing data. For logistical reasons, only kinetics and not kinematics and EMG were recorded for the jumps in the SJS.

### Centrifuge

The short-arm human centrifuge has a radius of 3.8m. The subject lies on a sled that can slide along two radial rails with minimal friction, as the only contact point between sled and rails is made via ball-bearings. The sled is fixed at the rails, with the fixation points at waist height serving as a rotational axis, allowing pitch, see Fig 2. The rotation direction of the centrifuge was always counter-clockwise. The rotational speed of the centrifuge was adjusted to achieve the desired acceleration at the center of mass. For example, an acceleration of 0.5g for a subject with a weight of 750N was achieved by setting a rotational speed that resulted in a summed ground reaction force of 750Nx0.5 = 325N when the subject was in a supine position with hips and knees extended.

### Sledge jump system

The SJS (see Fig 2) was described elsewhere in detail [9–11]. Briefly, the subject can jump in the horizontal plane with hardly any restrictions concerning the joint movements, allowing almost natural jumps. Since the movement direction is along the horizontal axis, the nearly constant forces generated by the two low-pressure cylinders substitute the gravitational force. The pressure was adjusted in a way that the forces produced by the cylinders matched the designated fraction of the subject’s weight, e.g. an acceleration of 0.5g for a subject with a weight of 750N was achieved by setting the force exerted by the low-pressure cylinders to 750Nx0.5 = 325N. In contrast to the centrifuge, the force acting on the jumper is the same for the whole body, and since the system does not rotate, there is no Coriolis effect involved. The position of the sledge was recorded via an incremental encoder.

### Kinematics

**Fig 3:**
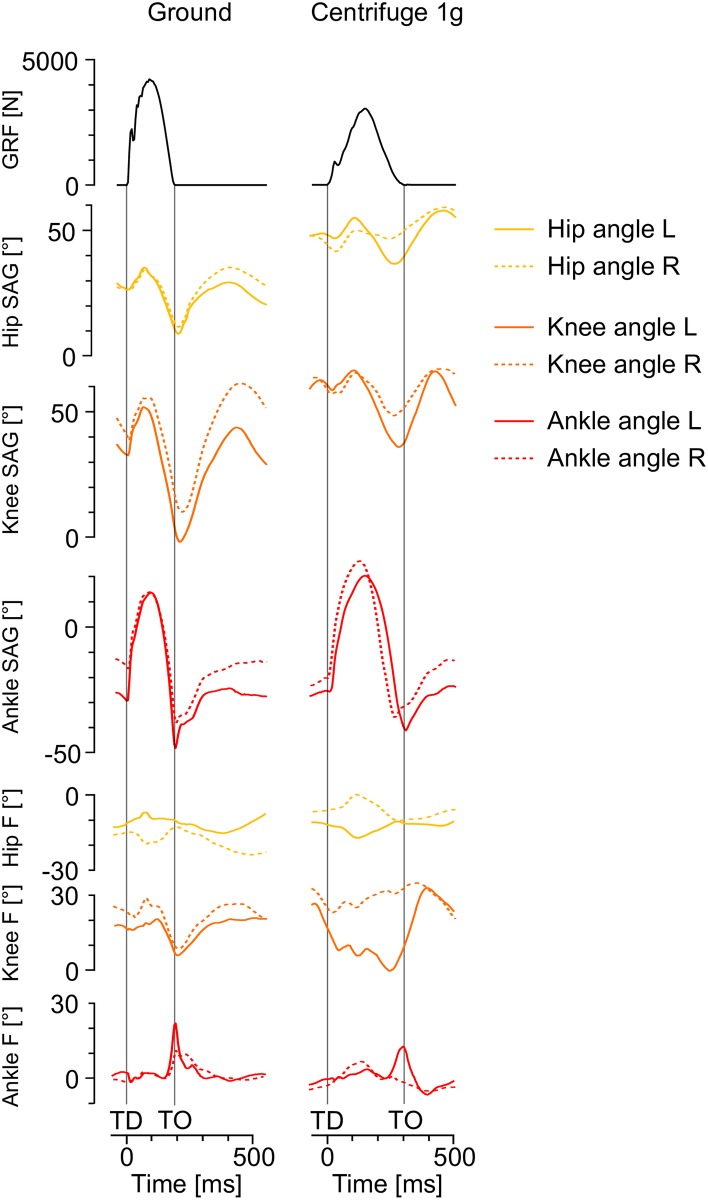
Exemplary data (ground reaction forces and joint angles) from one subject, on the left for vertical jumps on the ground, on the right for jumps in the centrifuge with 1g. Top to bottom: peak ground reaction force (combined from right and left force plates), hip angle in the sagittal plane (flexion-extension, solid line: left hip, broken line: right hip), knee angle in the sagittal plane, ankle angle in the sagittal plane, hip angle in the frontal plane, knee angle in the frontal plane, and ankle angle in the frontal plane. The vertical lines mark touchdown (TD) and take-off (TO). Zero values refer to neutral joint angles (hip and knee extended, ankle at 90°), positive values in the sagittal plane denote flexion, and positive values in the frontal plane denote adduction. Note the lower peak force, prolonged ground contact time, increased hip and knee flexion at touchdown and take-off as well as the smaller hip and knee amplitudes for the jumps in the centrifuge.

The jumps on the ground and in the centrifuge were recorded with a motion capturing system (Vicon, UK) using eight cameras (Bonita, 200 Hz). For the centrifuge measurements, the cameras were attached to the structure of the centrifuge, rotating together with the participant. As required by the plug-in gait model, the markers were placed on the following anatomical landmarks (left and right): anterior superior iliac spine, posterior superior iliac spine, thigh, medial and lateral knee joint center, shank, tibia, lateral and medial malleolus, heel, fifth metatarsal bone, hallux. Those markers were used to generate a 3D-model of the lower body, from which three joint angles were calculated (ankle, knee and hip). For exemplary data, see Fig 3. Together with the kinetic data, the kinematic data was used to calculate the knee moments using the Golem model [12].

### Electromyography

Wireless surface electrodes (Trigno, Delsys, USA) were placed over M. soleus (SOL), M. gastrocnemius lateralis (GL), M. rectus femoris (RF) and M. vastus lateralis (VL) of both legs. The longitudinal axes of the electrodes were in line with the presumed direction of the underlying muscle fibers. Interelectrode resistance was reduced by means of shaving and light abrasion of the skin. The EMG signals were wirelessly transmitted to the base station (band-pass filter 20Hz to 450Hz, signal gain of 909), sampled with 1 kHz and synchronized to the kinetic and kinematic data using the motion capturing system’s data acquisition unit (Vicon Giganet).

### Data processing

After removing DC offsets, the EMG signals were rectified. Afterwards, the means of the EMG and force signals were calculated for each trial, using the GRF as a trigger signal for the moment of touchdown (TD). Then, the mean amplitude voltage (MAV) was calculated by integrating the mean EMG signal during the preactivity phase (PRE, 50ms before touchdown until touchdown) as well as during the ground contact phase (GC, touchdown until takeoff). For the comparisons between gravity levels, the centrifuge EMG data was normalized to the ground EMG data, separately for each leg. The average rate of force development (RFD) was calculated as the peak force divided by the time from TD until the force signal reached its peak. The joint angles were determined at the time of TD and TO and the angular joint excursions were calculated from TD until the GRF reached its peak, i.e., for the eccentric phase of the jump, and from peak force until TO, i.e., the concentric phase of the jump. The leg stiffness was calculated according to Günther and Blickhan [13] as the ratio of the peak GRF and the displacement of the hip markers (greater trochanter) during the time interval from TD until the GRF reached its peak. Jump height was determined as the peak-to-peak displacement of the ankle markers in the direction of the jump (average of left and right lateral malleolus). Knee joint moments were calculated using inverse dynamics via the Vicon Golem model.

### Statistics

Group data are presented as means ± standard deviations (SD), calculations were done with JASP 0.84 (University of Amsterdam). The influence of the five different acceleration levels during centrifugation was assessed via analyses of variance for repeated measures, with acceleration level (five levels, 0.5g, 0.75g, 1g, 1.25g and 1.5g) and side (two levels, left and right) as within-subject factors. Differences between jumps with 1g in the centrifuge and normal jumps on the ground were assessed with two-tailed paired t-tests, as were differences between jumps in the centrifuge and their counterparts in the sledge jump system at the respective g-level.

## Results

The main result of the study was that the neuromuscular system is able to adapt rather complex full-body movements such as repetitive jumps to a non-constant force field and Coriolis forces. However, important kinetic and kinematic parameters such as peak forces, ground contact times and joint angles differed significantly between jumps in the centrifuge and jumps in the SJS and normal jumps on the ground, especially with lower g-levels.

### Kinetics

**Fig 4:**
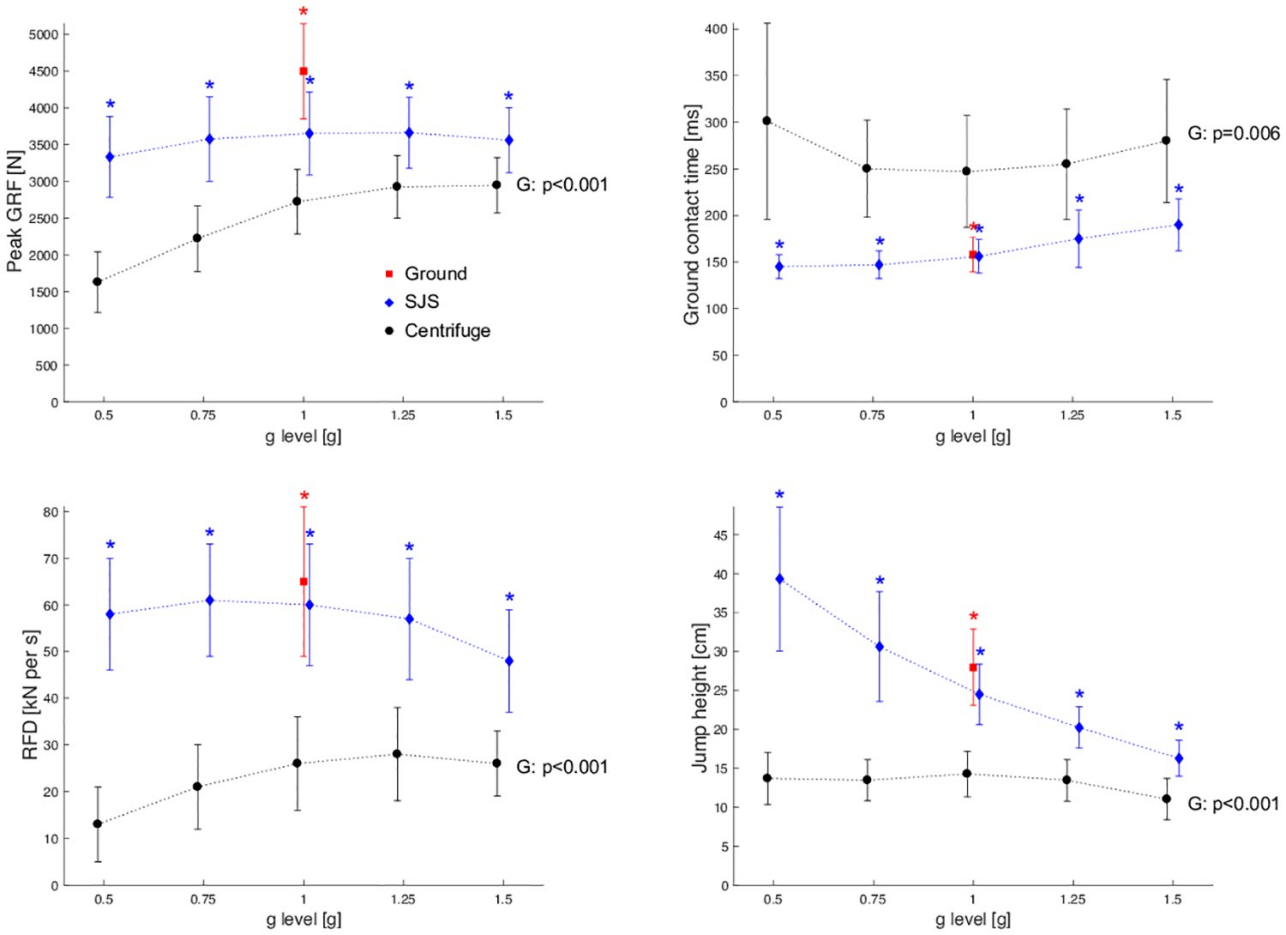
Grand mean of the ground reaction forces (GRF), ground contact times, rate of force development (RFD) and jump height. Black dots represent the hops in the centrifuge, blue dots the hops in the sledge jump system (SJS), and red dots the vertical hops on the ground. Red asterisk symbols denote a significant difference between hops on the ground and hops in the centrifuge with 1g, and blue asterisk symbols denote a significant difference between hops in the SJS and hops in the centrifuge at the respective g-level.

Even though for the higher g-levels the kinetic parameters came closer to the ones recorded in the SJS and during normal jumps, they were still significantly different. Peak ground reaction forces during centrifugation were 30–50% lower compared to vertical jumps, ground contact times were 60–90% longer, and RFD 50–80% lower, see Fig 4.

The variation of the acceleration from 0.5g to 1.5g was associated with a significant increase in peak GRF (+81%, p<0.001) and rate of force development (+93%, p<0.001). The ground contact time was prolonged in particular for the low (0.5g) and high (1.5g) g-levels, see Fig 4. Due to the counter-clockwise direction of rotation, the touchdown as well as the takeoff of the right leg during centrifugation was earlier than touchdown and takeoff of the left leg, see Table 1.

**Table 1:** Kinematics.

	AG 0.5	AG 0.75	AG 1.0	Ground	AG 1.25	AG 1.5	ANOVA
Hip excursion ECC SAG [°]	L: -3±4	-4±4	-3±5	0±4 p = 0.08	-3±4	0±5	G: **p<0.001**
	R: -2±5	-3±6	-1±6	0±3 p = 0.38	0±5	3±4	L/R: **p = 0.02**
Knee excursion ECC SAG [°]	L: 1±7	1±5	4±8	9±6 **p = 0.04**	5±6	7±7	G: **p<0.001**
	R: 0±8	1±8	4±8	9±6 **p = 0.04**	6±7	8±6	L/R: p = 0.99
Ankle excursion ECC SAG [°]	L: 28±9	31±7	34±8	36±7 p = 0.09	33±6	32±6	G: **p<0.001**
	R: 28±9	32±8	35±8	35±7 p = 0.85	37±6	36±6	L/R: p = 0.14
Hip angle at TD FRONT [°]	L: -9±4	-9±3	-9±3	-9±3 p = 0.78	-9±3	-8±3	G: p = 0.92
	R: -5±3	-5±4	-5±4	-7±4 **p = 0.01**	-5±3	-5±3	L/R: **p = 0.02**
Hip excursion ECC FRONT [°]	L: -1±1	-2±1	-2±2	0±1 **p<0.001**	-2±2	-2±2	G: p = 0.61
	R: 3±1	4±1	4±2	1±2 **p<0.001**	4±2	4±2	L/R: **p<0.001**
Hip excursion CON FRONT [°]	L: 1±1	0±3	1±3	0±3 p = 0.24	1±4	1±3	G: p = 0.25
	R: -4±1	-4±2	-4±2	0±3 **p = 0.004**	-4±2	-3±2	L/R: **p<0.001**
Knee angle at TD FRONT [°]	L: 14±8	14±8	15±10	12±8 **p = 0.002**	16±9	16±10	G: **p = 0.03**
	R: 11±11	10±12	11±13	10±9 p = 0.74	12±11	13±11	L/R: p = 0.17
Knee excursion ECC FRONT [°]	L: -2±3	-3±4	-2±6	2±3 **p<0.001**	-2±6	0±5	G: p = 0.13
	R: -1±4	0±4	1±5	3±3 p = 0.26	1±4	2±6	L/R: **p = 0.03**
Knee excursion CON FRONT [°]	L: 1±3	0±3	-1±6	-9±7 **p<0.001**	-4±5	-4±6	G: **p<0.001**
	R: 3±2	1±5	0±5	-8±7 **p<0.001**	-2±7	-2±8	L/R: p = 0.24
Ankle angle at TD FRONT [°]	L: 1±7	1±6	1±6	1±5 p = 0.45	0±5	-1±5	G: p = 0.22
	R: 1±7	1±7	0±6	1±4 p = 0.32	0±5	1±6	L/R: p = 0.73
Ankle excursion ECC FRONT [°]	L: -1±5	0±6	0±7	2±7 **p = 0.009**	3±7	3±7	G: **p<0.001**
	R: -1±7	-1±7	1±8	1±5 p = 0.87	1±6	1±8	L/R: p = 0.75
Ankle excursion CON FRONT [°]	L: 2±8	3±10	2±10	7±12 **p = 0.02**	1±10	-1±11	G: **p = 0.03**
	R: 5±12	7±14	6±14	6±10 p = 0.88	7±13	4±11	L/R: p = 0.1
TD side difference [ms]	12±16	9±9	10±15	0±4 **p = 0.02**	10±12	7±15	G: p = 0.52
TO side difference [ms]	17±26	15±24	19±31	0±4 **p = 0.03**	15±29	17±34	G: p = 0.79
Leg stiffness [kN/m]	29±13	33±10	35±10	43±11 **p = 0.03**	35±9	33±6	G: p = 0.053

Mean and standard deviation of the joint angle excursions during the eccentric phase of the jumps (ECC, from touchdown until peak force) as well as the concentric phase (CON, from peak force until take-off) in the sagittal plane (SAG) and the frontal plane (FRONT), as well as the joint angles during the moment of touchdown (TD), as well as the leg stiffness during the eccentric phase and the side differences at touchdown and take-off (TO), i.e., the time differences between the touchdown or take-off of the right foot and the left foot. A negative value for a joint angle means that the joint is extended (SAG) or abducted (FRONT) relative to its neutral position. Similarly, a negative value for a joint excursion means that the joint is extended (SAG) or abducted (FRONT) during that phase. The 1g centrifuge condition is compared to the ground condition via two-tailed paired t-tests (p-values in the ground column), and the influence of gravity level (G) as well as side differences (L/R) are analysed via rmANOVAs (last column).

### Kinematics

**Fig 5:**
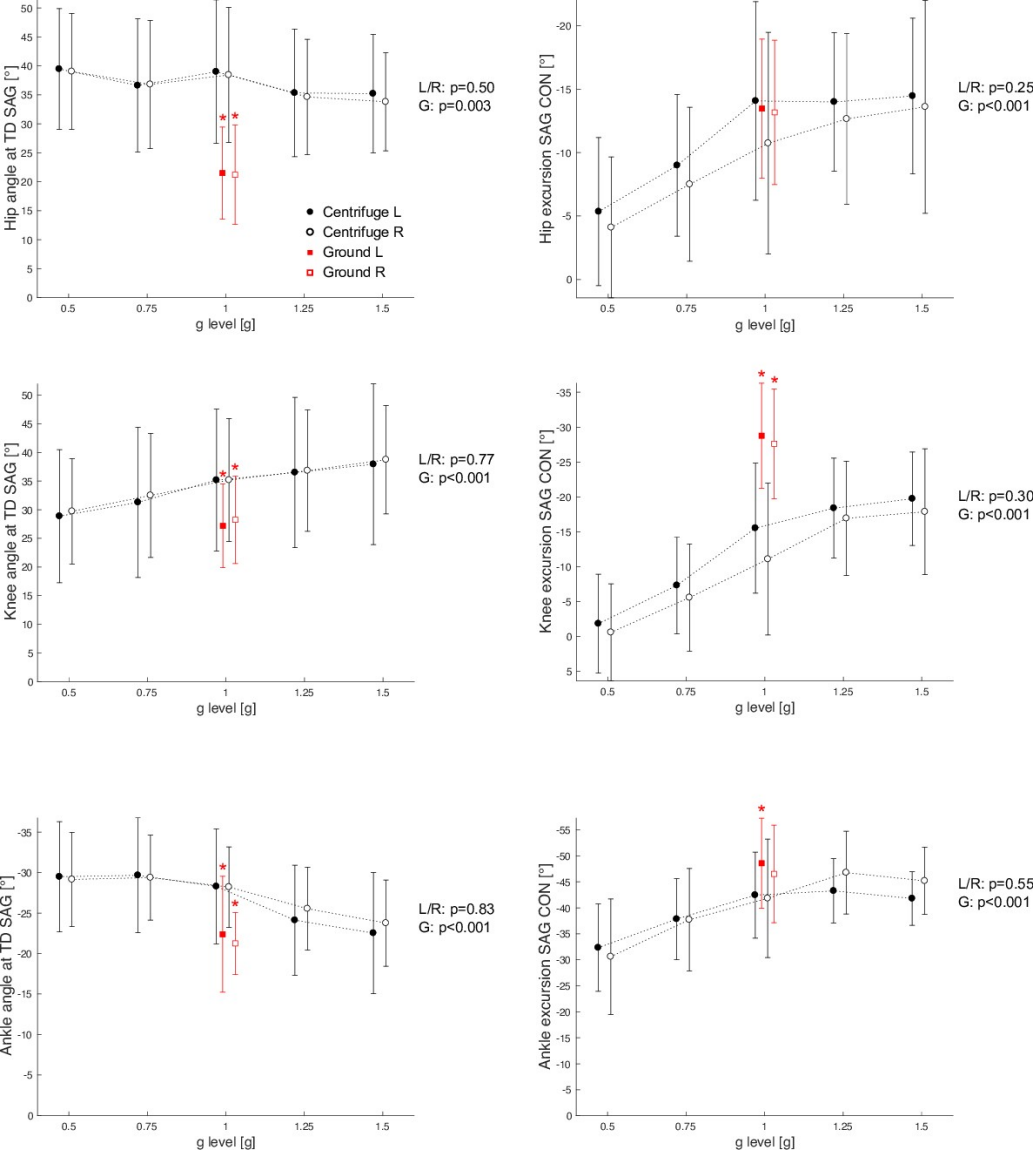
Grand mean of the joint angles in the sagittal plane (SAG) at touchdown (TD), as well as the joint excursions during the concentric phase (CON) of the jump, i.e., the joint amplitudes between the time when the GRF reached its peak and take-off. Solid circles represent the left hip, knee and ankle angles of the hops in the centrifuge, open circles represent the right joint angles of the hops in the centrifuge. Solid squares represent the left joint angles of the hops on the ground, open squares represent the right joint angles of the hops on the ground. A negative value for a joint angle means that the joint is extended relative to its neutral position. Similarly, a negative value for a joint excursion means that the joint is extended during that phase. Asterisk symbols denote a significant difference between hops on the ground and hops in the centrifuge with 1g. No significant side differences were observed (main effect of side in the ANOVA, L/R p-values), but significant effects of gravity (main effect of gravity, G p-values).

The most pronounced kinematic differences were observed for jump height and knee and hip joint angles, whereas the ankle joint angles were more comparable. During centrifugation, jump height was 50–60% lower, and hip and knee joints were considerably more flexed at both touchdown and takeoff, with reduced joint excursions, see Fig 5 and Table 1. Some of the differences in the frontal plane were more pronounced for one side, reflected by significant main effects of side.

Significant differences as a result of the acceleration variation were observed for most of the kinematic parameters: jump height decreased, at touchdown hip flexion and ankle plantar flexion decreased and knee flexion increased, all sagittal-plane joint excursions increased in both the eccentric and concentric phase, frontal-plane knee adduction at touchdown and knee abduction during the concentric phase increased, and ankle excursions changed during both phase, see Fig 5 and Table 1. No significant differences due to the variation of the acceleration were observed for leg stiffness, the frontal-plane hip angles and excursions, the frontal-plane knee excursion during the eccentric phase, and the frontal-plane ankle angle at touchdown.

### Joint moments

**Fig 6:**
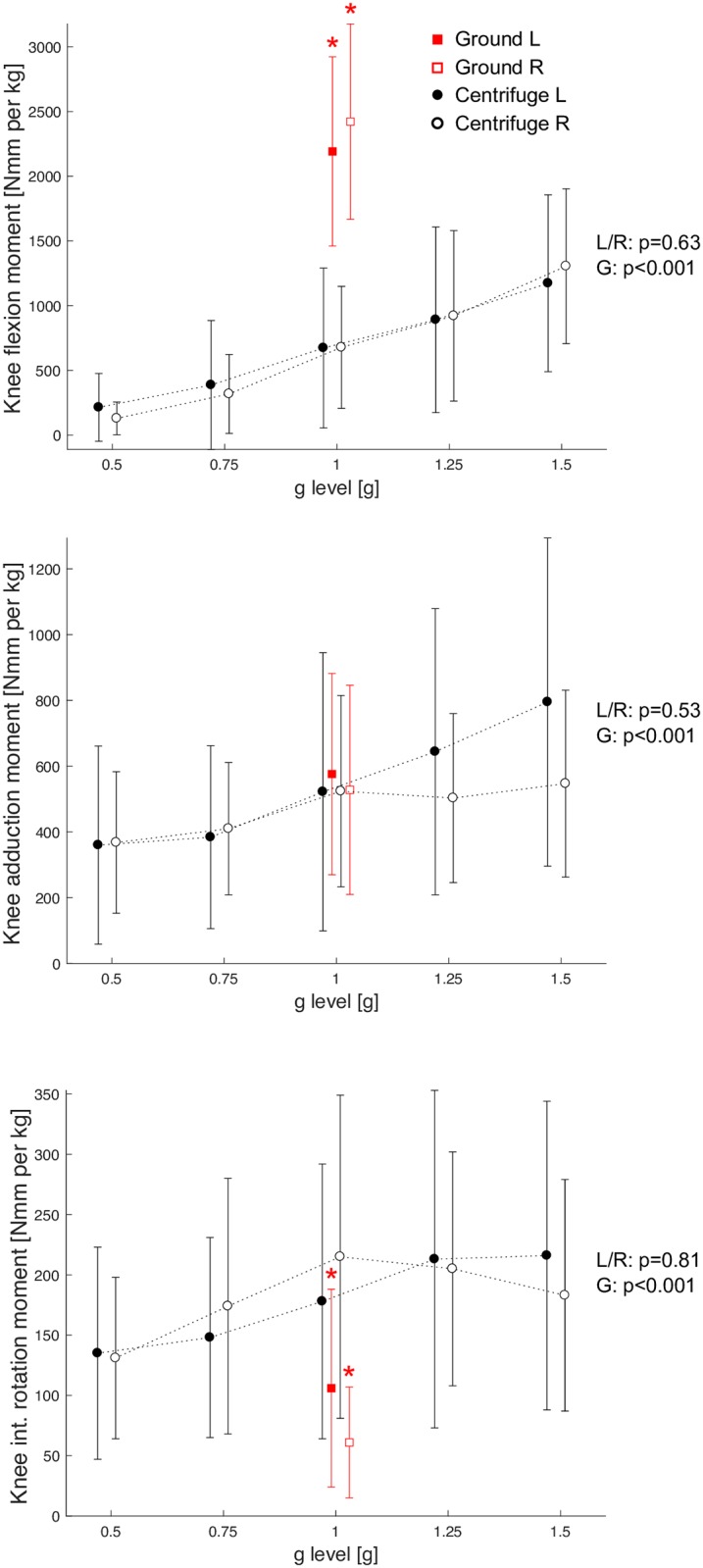
Grand mean of the peak knee moments (flexion, adduction and internal rotation), normalized to body mass. Solid circles represent the left knee moments of the hops in the centrifuge, open circles represent the right knee moments in the centrifuge. Solid squares represent the left knee moments of the hops on the ground, open squares represent the right knee moments on the ground. Asterisk symbols denote a significant difference between hops on the ground and hops in the centrifuge with 1g. No significant side differences were observed (main effect of side in the ANOVA, L/R p-values), but increased moments with increasing gravity level (significant main effect of gravity, G p-values).

The knee joint moments increased with increasing acceleration level. Knee flexion moments were significantly lower during centrifugation when compared to vertical jumps, whereas adduction moments were comparable and internal rotation moments were higher, see Fig 6.

### Muscle activity

**Fig 7:**
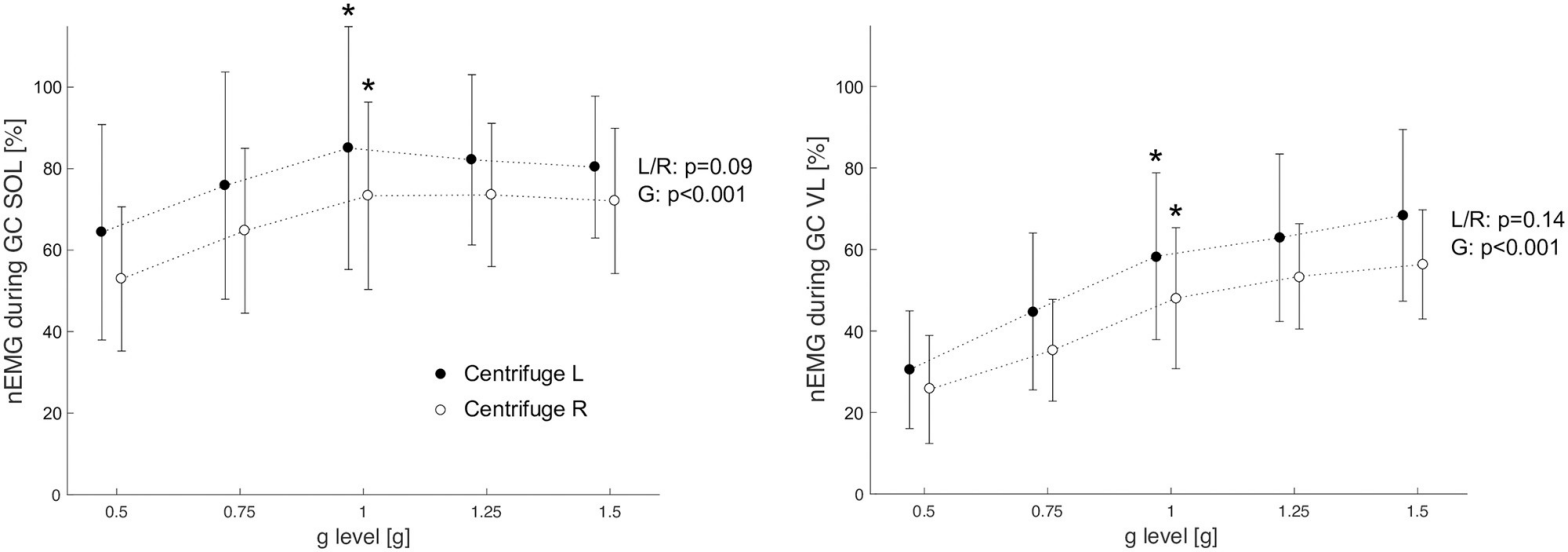
Grand mean of the normalized electromyographic activity (nEMG) during the ground contact phase of the soleus muscle (SOL) and the vastus lateralis muscle (VL). Values are mean amplitude voltages of the hops in the centrifuge normalized to the mean amplitude voltages of the hops on the ground, i.e., a value of 100% means that the EMG activity is the same in the centrifuge as on the ground. Solid circles represent the EMG of the left muscles, open circles represent the EMG of the right muscles. Asterisk symbols denote a significant difference between hops on the ground and hops in the centrifuge with 1g. Muscle activity in the left leg tended to be higher than in the right leg (not significant this case, main effect of side in the ANOVA, L/R p-values), and increased with gravity level (main effect of gravity, G p-values).

During the preactivity phase—the 50ms before touchdown—as well as during the ground contact phase, the muscle activity (mean amplitude voltage, MAV) of the leg extensors showed an increase in response to increasing acceleration levels, but was significantly lower compared to normal jumps on the ground, with the exception of the lateral gastrocnemius muscle, see Table 2 and Fig 7. Muscle activity of the leg extensors tended to be higher for the left leg than for the right leg, but the main effect of side was only significant during PRE for the knee extensors, see Table 2.

**Table 2:** Muscle activity.

	AG 0.5	AG 0.75	AG 1.0	Ground	AG 1.25	AG 1.5	ANOVA
RF PRE [%]	L: 25±13	34±15	45±30	0.064±0.030 **p<0.001**	49±30	56±47	G: **p<0.001**
	R: 18±10	20±11	25±16	0.065±0.027 **p<0.001**	30±17	38±20	L/R: **p = 0.002**
VL PRE [%]	L: 33±21	53±41	61±45	0.132±0.064 **p = 0.007**	55±34	54±34	G: **p = 0.006**
	R: 28±23	40±31	43±32	0.132±0.082 **p = 0.004**	46±31	46±26	L/R: **p = 0.03**
GL PRE [%]	L: 70±46	82±44	103±81	0.101±0.051 p = 0.13	96±57	96±48	G: **p<0.001**
	R: 60±35	73±40	82±47	0.156±0.065 **p = 0.03**	85±34	84±29	L/R: p = 0.28
Sol PRE [%]	L: 29±20	38±25	44±19	0.076±0.027 **p<0.001**	52±31	58±30	G: **p = 0.007**
	R: 38±23	43±24	47±28	0.086±0.019 **p<0.001**	48±25	48±22	L/R: p = 0.94
RF GC [%]	L: 29±17	38±22	48±32	0.122±0.034 **p<0.001**	59±55	60±41	G: **p<0.001**
	R: 28±13	34±18	42±24	0.115±0.037 **p<0.001**	49±25	54±21	L/R: p = 0.28
VL GC [%]	L: 29±15	44±19	57±21	0.187±0.068 **p<0.001**	62±21	67±21	G: **p<0.001**
	R: 24±13	34±18	47±17	0.182±0.077 **p<0.001**	52±13	55±13	L/R: p = 0.14
GL GC [%]	L: 90±107	104±111	113±131	0.135±0.058 p = 0.17	107±93	93±54	G: p = 0.17
	R: 84±57	97±51	106±64	0.172±0.070 p = 0.41	99±38	94±37	L/R: p = 0.83
SOL GC [%]	L: 64±26	76±28	85±30	0.137±0.044 **p = 0.02**	82±21	80±17	G: **p<0.001**
	R: 53±18	65±20	73±23	0.128±0.038 **p = 0.002**	74±18	72±18	L/R: p = 0.09

Mean and standard deviation of the EMG activity of all participants for the four recorded muscles (rectus femoris, vastus lateralis, gastrocnemius lateralis, soleus) during two phases (PRE 50ms before touchdown until touchdown, and total activity during ground contact (GC) from touchdown until take-off). For comparison’s sake, the values of the hops in the centrifuge are normalized to the values of the hops on the ground, i.e., the centrifuge EMG is expressed in percent of the ground EMG, whereas the ground EMG is expressed as mean amplitude voltage in mV. The 1g centrifuge condition is compared to the ground condition via two-tailed paired t-tests (p-values in the ground column), and the influence of gravity level (G) as well as side differences (L/R) are analysed via rmANOVAs (last column).

## Discussion

The results show that in principle, complex full-body movements such as reactive jumps can be quickly adapted to a non-constant force field and varying acceleration levels. However, key parameters such as peak ground reaction force, rate of force development and ground contact time differed significantly from the ones observed during vertical jumps, especially for acceleration levels lower than 1g. The analyses of the jump height and the kinematic data suggest that especially for the lower gravity levels, participants used a modified movement strategy with more flexion in the hip and knee joints, reduced joint excursions, lower jump height and prolonged ground contact times, probably to reduce the limb deflections and other effects of the Coriolis force and the non-constant force field.

The assumption that the differences between jumps during centrifugation and vertical jumps stem primarily from a different movement strategy aimed at reducing the effects of Coriolis forces and the non-constant force field is supported by the observation that the jumps in the sledge jumps system were much more comparable to vertical jumps. Both in the sledge jump system and the centrifuge the participants performed the jumps in a supine position with gravity levels between 0.5g and 1.5g, but in the sledge jump system the force that is applied to the participant is constant and the system is not rotating. It is therefore straightforward to propose that the additional differences between jumps during centrifugation and jumps in the sledge jump system stem primarily from the different force fields. Indeed, the jump height in the SJS increased in a more or less linear fashion from 1g to 0.5g (25cm to 39cm on average), whereas it remained at about 14cm for the jumps in the centrifuge. The lower hip and knee excursions during centrifugation—especially for the lower gravity levels, with virtually no knee extension during the concentric push-off phase—also fit with a strategy aimed at reducing limb deflections. In contrast, this strategy has not been reported for horizontal jumps in the absence of rotation [10]. Concomitantly, the muscle activity of the leg extensors during centrifugation was greatly reduced, especially for the knee extensors and lower gravity levels, which amounted to only about 30% of the activity recorded for vertical jumps. In contrast, the EMG of the lateral gastrocnemius muscle was comparable during centrifugation and vertical jumps, and soleus activity during ground contact only reduced to about 80%, which is consistent with the observation that the ankle joint excursions were much more comparable than the knee and hip joint excursions. It is interesting to note that despite the clear kinetic and kinematic differences between the jumps in the centrifuge and the vertical jumps, leg stiffness was not much lower in the centrifuge and did not change much with increasing g-level, suggesting that leg stiffness might be an important parameter that is being kept quite constant by the neuromuscular system [9].

Possibly due to the adapted movement strategy with reduced joint excursions, side differences between left and right leg during centrifugation were smaller than expected. There were almost no side differences in the sagittal plane—i.e. in the flexion-extension joint angles and excursions—and the kinematic differences in the frontal plane were mostly limited to the hip joint, with more abduction in the left leg at touchdown and during the eccentric phase, and adduction during the concentric phase, which is consistent with a preemptive leg positioning to the left, aimed at counteracting the following leg deflection to the right that is caused by the Coriolis force during counter-clockwise rotation. When comparing the muscle activity between the left and the right leg, a higher activity of the left leg is apparent during centrifugation, especially in the knee extensors during the preactivity phase before touchdown. As touchdown of the right leg occurred before the touchdown of the left leg, the lower preactivity of the right leg might be aimed at having a more compliant right leg during the initial part of the right leg’s contact phase [14], thus "waiting" for touchdown of the left leg. A different explanation could be that due to the counter-clockwise rotation, the left leg has to withstand higher forces than the right leg.

Concerns that knee joint moments might be excessively high during centrifugation due to Coriolis forces and limb deflections seem to be rather unfounded: knee flexion moments were significantly lower during centrifugation, knee adduction moments comparable (albeit higher for the left leg and higher g-levels), only the internal rotation moments were significantly higher during centrifugation. From studies comparing vertical jumps and cutting maneuvers, it is known that jumps evoke relatively low knee adduction/abduction moments compared to cutting maneuvers [15] or pivot tasks [16], so even the slightly increased knee adduction moments observed in the left leg during higher g-levels should not entail a significantly increased risk of injury. Similarly, the increase in knee internal rotation moments during centrifugation is comparable to the increase reported for cutting maneuvers compared to vertical jumps [15], and pivot tasks [17].

From an applied perspective, it can be concluded that the combination of jumps as an effective countermeasure for the deteriorating effects of physical inactivity and artificial gravity as a promising countermeasure for the systemic effects of spaceflight is possible, albeit with lower peak forces and lower rate of force development. A g level of about 1g is recommended, as g levels below 1g lead to inferior results, and g levels above 1g do not seem to produce considerable benefits, but increase knee joint moments and might not tolerated as well, as anecdotal evidence from one participant with a pre-syncope at 1.5g suggests and literature confirms for higher g-levels [18]. Whether a longer familiarization period in the centrifuge would allow participants to develop a movement pattern that is closer to the natural one, resulting in higher peak forces, has to be answered in a future training study. Previous studies using rotating rooms and finger pointing tasks indicate that for isolated movements and slow rotation speeds, adaptation to the Coriolis force perturbations of the arm trajectory was complete after 40 repetitions [19], but the results of the present study suggest that for dynamic whole-body movements such as jumps, one familiarization session consisting of more than 200 jumps does not seem to be sufficient (continuous improvements were visible both during the familiarization session and the test session).

In conclusion, an adaptation to acceleration levels other than the normal constant gravitational acceleration of 1g is possible, even in the presence of a non-constant force field and Coriolis forces. However, centrifugation introduced additional constraints compared to a constant force field without rotation, resulting in lower peak forces and changes in kinematics. These changes can be interpreted as a movement strategy aimed at reducing lower limb deflections caused by Coriolis forces.
